# Moving Beyond the Pillars of Cancer Treatment: Perspectives From Nanotechnology

**DOI:** 10.3389/fchem.2020.598100

**Published:** 2020-11-10

**Authors:** Cerise M. Siamof, Shreya Goel, Weibo Cai

**Affiliations:** ^1^Department of Biochemistry, University of Wisconsin-Madison, Madison, WI, United States; ^2^Department of Materials Science and Engineering, University of Wisconsin-Madison, Madison, WI, United States; ^3^Department of Radiology, University of Wisconsin-Madison, Madison, WI, United States; ^4^Department of Medical Physics, University of Wisconsin-Madison, Madison, WI, United States; ^5^Department of Pharmaceutical Sciences, University of Wisconsin-Madison, Madison, WI, United States

**Keywords:** drug delivery, radiation therapy, immunotherapy, theranosctics, nanomedcine

## Abstract

Nanotechnology has made a significant impact on basic and clinical cancer research over the past two decades. Owing to multidisciplinary advances, cancer nanotechnology aims to address the problems in current cancer treatment paradigms, with the ultimate goal to improve treatment efficacy, increase patient survival, and decrease toxic side-effects. The potential for use of nanomedicine in cancer targeting and therapy has grown, and is now used to advance the four traditional pillars of cancer treatment: surgery, chemotherapy, radiation therapy and the newest pillar, immunotherapy. In this review we provide an overview of notable advances of nanomedicine in improving drug delivery, radiation therapy and immunotherapy. Potential barriers in the translation of nanomedicine from bench to bedside as well as strategies to overcome these barriers are also discussed. Promising preclinical findings highlight the translational and clinical potential of integrating nanotechnology approaches into cancer care.

## Introduction

Cancer is a leading cause of death globally, with 18 million cases and 9 million deaths worldwide each year (Bray et al., 2018). Despite a promising decline in mortality rates over the past 10 years, more than half a million people die annually of cancer in the United States alone (Miller et al., 2019; Siegel et al., 2019). Traditional cancer treatment options can be classified into distinct pillars: surgery, chemotherapy, radiation therapy (hereon referred to as external radionuclide therapy, or ERT) and a more recently added fourth pillar; immunotherapy. Decades of concerted efforts have radically transformed the face of clinical cancer care and has identified specific weaknesses in each of these pillars that are now being targeted by designer personalized therapies aimed at improving survival rates and reducing treatment side-effects.

Since the first FDA approval of the liposomal doxorubicin (Doxil) in the 1990s (Grodzinski et al., 2019), nanomedicine approaches have emerged as a formidable means to improve the outcomes of traditional pillars of cancer therapy, each of which has its own set of advantages and disadvantages. Surgery is by nature more invasive than other treatment options, but can be used as a frontline treatment for primary tumor masses, for example in cases of prostate cancer (Petrelli et al., 2014). Of course, the location of tumor site must be known for surgery to be effective, which is not always possible, especially in cases where cancers have become invasive or metastasized to different organs. Smart nanomedicine can enhance efficacy of traditional surgical procedures through advances in lymph node mapping (Ravizzini et al., 2009; Erogbogbo et al., 2011; Rubio et al., 2015) and intraoperative image-guided surgery to achieve complete oncological resection (Bradbury et al., 2013; Zheng et al., 2015; Sun et al., 2017). Further, novel approaches such as NP-mediated phototherapy of non-resectable or residual tumor margins can potentially improve curative rates in several cancer types, for example, thoracic malignancies (Keereweer et al., 2011; Bradbury et al., 2013; Lee et al., 2015; Locatelli et al., 2015; Hofferberth et al., 2016; Owens et al., 2016). Theranostic nanomedicine (combination of diagnostic and therapeutic entities into a single platform) can potentially improve outcomes in the post-operative settings as well (Feng et al., 2020). Synergism of precision surgery and nanomedicine has been explored in depth in other excellent reviews, and thus the intersection of nanomedicine with surgical oncology is not a main focus of this review (Singhal et al., 2010; Wang et al., 2019).

By contrast, chemotherapy, ERT and immunotherapy are minimally invasive options, and chemotherapy in particular is now a hallmark of modern cancer treatments (Schirrmacher et al., 2003). However, basic ERT and chemotherapy often pose the risk of damage to benign body cells, causing toxicity and undesirable side effects to the patient, accompanied by very modest treatment outcomes. In regards to the problem of non-specificity with chemotherapy, advances in the multidisciplinary fields of chemistry, biomedical engineering, materials sciences, biophysical, and biochemical sciences have enabled development of novel targeted therapies to improve drug formulations and delivery, as well as overcoming drug resistance (Peer et al., 2007). ERT has benefitted from multidisciplinary advances in irradiation techniques and effective nanoscale radiosensitizers that ensure accurate dose distributions that spare normal tissues. Immunotherapy, on the other hand, has shown mixed results, with efficacy varying drastically from patient to patient and among different cancer types. Nanotechnology has benefitted immunotherapy through improved delivery of immunomodulatory compounds that induce local/systemic anti-tumor immunity or have a tumor priming effect (Martin et al., 2020). Furthermore, high-performance combinations of these fundamental pillars themselves or with other emerging treatment modalities afforded by nano-engineering promises significant implications across preclinical and clinical settings (Kobayashi et al., 2010).

In this work, we provide an overview of the recent research advances in the field of nanotechnology that have dramatically impacted the pillars of cancer treatment, and discuss the opportunities and challenges in these emerging areas. We begin by reviewing advances in targeted drug delivery system, focusing on the use of NPS in stimuli-responsive chemotherapy, such as pH, enzyme, ROS, and hypoxia-sensitive systems. We then move to another traditional pillar of cancer treatment: ERT, and review the uses of nanotechnology within ERT, paying special attention to the ability of nanotechnology to combine ERT with other types of therapy, including both chemotherapy and immunotherapy. Lastly, we review exciting advances in the newest pillar: immunotherapy, describing how nanotechnology may improve therapies targeted to both the innate and adaptive immune system, including nanovaccines, innate immune cell-activation, and immune checkpoint inhibition.

## Targeted Chemotherapy and Drug Delivery Systems

The inability of traditional chemotherapy drugs to distinguish cancer from self and suboptimal pharmacokinetics, pose several complications to cancer treatment. Chemotherapy can result in cardiomyopathy (Shakir and Rasul, 2009; Kumar et al., 2012; Higgins et al., 2015), neuropathy (Kannarkat et al., 2007; Windebank and Grisold, 2008), and nephrotoxicity (Weiss and Poster, 1982; Hanigan and Devarajan, 2003), causing significant concerns for patient morbidity and mortality. NPs have a high surface area to volume ratio which makes them efficient in use for drug loading and delivery (Singh and Lillard, 2009) and have been recognized as a promising approach to selectively target the tumor site by passive [enhanced permeability and retention (EPR) effect (Torchilin, 2011)] or active targeting (Byrne et al., 2008) approaches, to reduce normal tissue uptake and undesirable side-effects of chemotherapeutics. Passive targeting exploits characteristic features of tumors, particularly leaky vasculature, to enhance the accumulation of drug-loaded NPs in the tumor. Active targeting approaches achieve enhanced drug delivery by conjugating drug-loaded NPs with moieties that specifically bind to receptors overexpressed on target cells, such as proteins, polysaccharides, and other small molecules (Yoo et al., 2019). Several recent reviews describe these paradigms in detail (Byrne et al., 2008; Bazak et al., 2015). Furthermore, external stimuli such as temperature, light and magnetically-guided delivery and release of chemotherapeutics are emerging strategies that promise significant advances in targeted drug delivery (Dai et al., 2017). For more in depth review stimuli-responsive drug delivery systems, readers are referred to other more detailed reviews (Ruoslahti et al., 2010; Mura et al., 2013; Zhou L. et al., 2018).

Despite being promising, the final outcomes of such strategies are severely influenced by the intrinsic physiological factors within the tumor. Hence, smart nanomedicine approaches have focused on developing NPs carefully engineered to specifically harness the unique tumor microenvironment (TME) to increase the specificity and efficacy of the treatment. There are several distinct physiological features of the TME that can be exploited by NPs for improved chemotherapy outcomes, including acidic pH, reactive oxygen species (ROS), overexpression of certain enzymes, and lack of intratumoral oxygen or hypoxia. In the following sections, we highlight promising TME-responsive NPs that offer a universal approach for anti-cancer therapy by targeting the genral physiological abnormalities found in all tumors.

### pH-Responsive Nanosystems

The body varies considerably in pH: physiological pH is ~7.4, while that at the tumor site ranges from pH 5.7–7.8 (Gao et al., 2010), due to altered metabolism (glycolysis) and hypoxia resulting in lactic acid formation (Feron, 2009; Danhier et al., 2010). This known physiological difference allows for the development of pH-sensitive nanocarriers that can release their cargo in a more targeted fashion. These systems generally rely on change in structure or size upon exposure to acidicity, or the breaking of a bond sensitive to the acidic pH, which allows for controlled release of the cargo drug at the tumor site (Gao et al., 2010; Tao et al., 2018). pH sensitivity can be conferred to NPs through the use of acid-labile moieties (Li et al., 2016). For example, platinum pro-drug conjugated polymeric NPs form large (~100 nm) nanoclusters (NC) in physiological pH that enhances their accumulation at the tumor site through prolonged blood circulation and EPR effect (Li et al., 2016). On exposure to acidic pH, an amide bond in cleaved, and NCs release small polyamidoamine prodrug dendrimers (~ 5 nm) that facilitates greater tumor penetration and cellular internalization. Another redox-responsive moiety that has been used to respond to high concentrations of glutathione is poly(disulfide amide) (PDSA) (Kong et al., 2019). Other examples of pH sensitive moieties used to develop acidosis-responsive NPs include acetals, hydrazones, anhydrides, and Schiff bases (He et al., 2013; Zhang et al., 2016).

**Figure 1:**
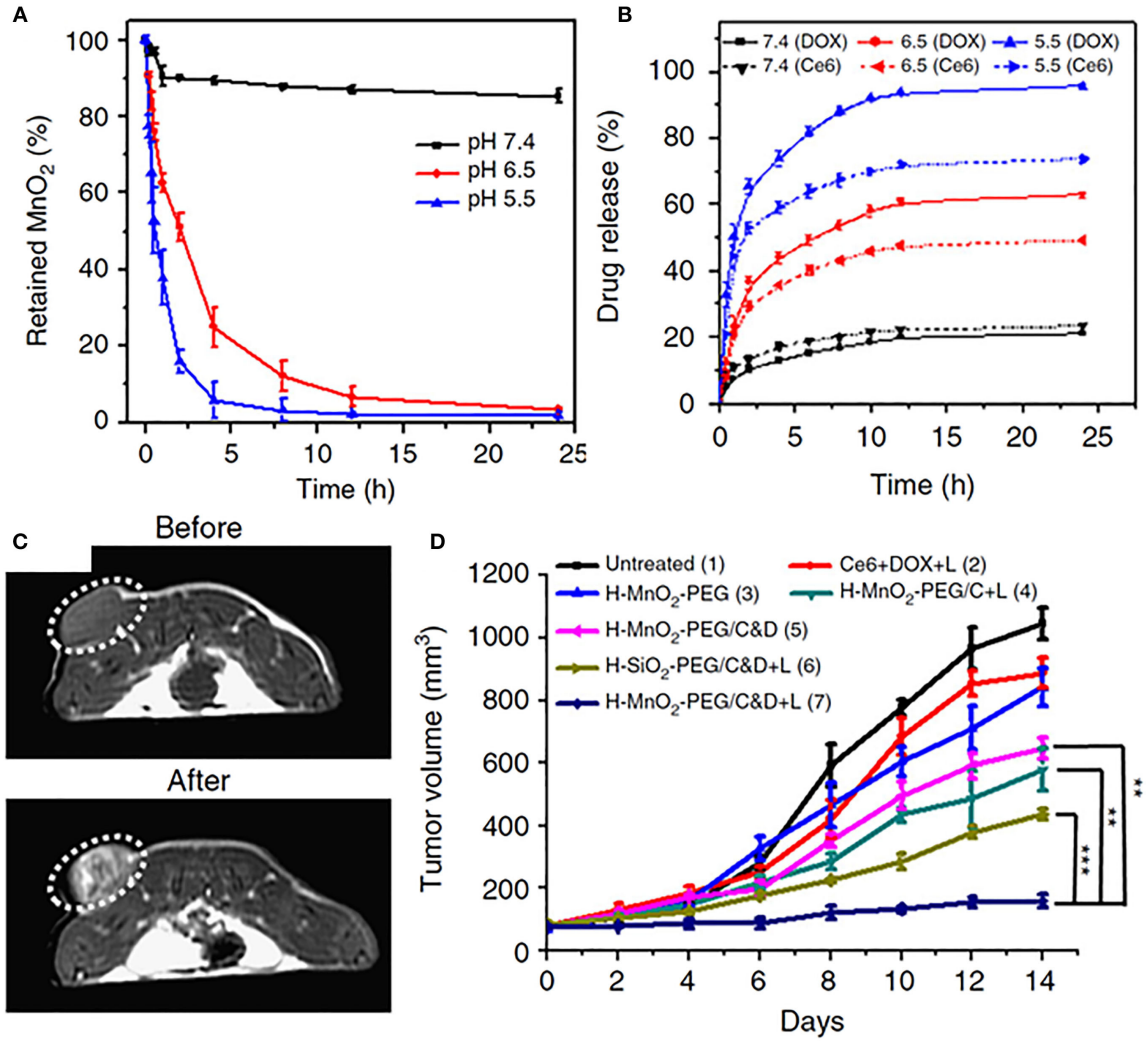
**(A)** pH-dependent nanoparticle decomposition of hollow MnO_2_-PEG nanoparticles dispersed in solutions of different pH (7.4, 6.5, and 5.5) determined by the absorbance of MnO_2_. **(B)** Percentages of released Ce6 and DOX from H-MnO_2_-PEG/C&D over time in the presence of 10% fetal bovine serum (FBS) at different pH values. **(C)** *In vivo* axial T1-MR images of a 4T1- tumor bearing mouse taken before and 24 h post i.v. injection of H-MnO_2_-PEG/C&D. **(D)** Tumor growth curves in different groups after various treatments as indicated. Chemo-PDT treatment after injection of pH-responsive H-MnO_2_-PEF/C&D nanoparticles depicted most significant reduction in tumor volumes. With permission from Yang et al. (2017). *p* values were calculated by Tukey's post-test (***p < 0.001, **p < 0.01, and *p < 0.05).

Multifunctional inorganic NPs have also been designed for pH-responsive imaging and drug delivery applications. For example, Yang et al. reported a novel biodegradable hollow MnO_2_ nanocarrier (H- MnO_2_) co-loaded with chemotherapeutic doxorubicin (DOX) and a photosensitizer chlorin e6 (Ce6), utilizing pH sensitivity for both specific imaging and on-demand drug release (Yang et al., 2017). The NPs were stable at neutral pH but exhibited time-dependent degradation behavior in increasingly acidic pH, from 6.5 to 5.5 (Figure 1A), resulting in enhanced release of both DOX and Ce6 (Figure 1B). pH-responsiveness of the NPs derived from reaction with either H^+^ ions or glutathione present in the TME. H- MnO_2_ NPs enabled tumor-specific magnetic resonance (MR) imaging (Figure 1C) as well as efficient drug release, which translated to greatly reduced tumor burdens when coupled with Ce6-enabled photodynamic therapy (PDT) (Figure 1D). Of note, H-MnO_2_ triggered hypoxia alleviation in the tumor resulted in enhanced combination chemo-PDT efficacy as well as reversal of immunosuppressive TME. When further combined with immune checkpoint inhibitors, the pH-responsive chemo-PDT by H-MnO_2_ demonstrated effective abscopal effect by not only inhibiting the growth of primary tumors, but also of distant tumor sites that were not irradiated with the laser. The work highlights a multi-pronged approach to tumor eradication by pH responsive NPs and makes a compelling case for exploring experimental therapeutic approaches in conjunction with the established paradigms for more effective outcomes, that is desirable for both researchers and patients.

### Enzyme-Sensitive Nanosystems

The overexpression of different enzymes in tumors has been exploited to develop smart microenvironment-responsive nanomedicine. Tumors are characterized by elevated levels of enzymes such as galactosidases, phospholipases, cathepsins, and matrix metalloproteinases (MMPs) (Kessenbrock et al., 2010; Cal and López-Otín, 2015; Zhang et al., 2019). MMPs in particular, have been widely harnessed to develop enzyme-responsive drug delivery systems owing to their involvement in signaling pathways important for tumor cell growth and migration and apoptosis (Kessenbrock et al., 2010). Conveniently, one substrate of MMPs is gelatin, which is biocompatible and non-immunogenic (Xu et al., 2013). Exploiting this, the authors prepared mesoporous silica nanoparticles (MSNs) which were coated with a gelatin matrix, then loaded with DOX (MSN-Gel-DOX) (Xu et al., 2013). The gelatin matrix prevented premature release of DOX, and on exposure to MMP-9 in the TME, was degraded, allowing for enhanced DOX release. This was confirmed *in vitro* where MMP-9 triggered increased DOX release from the MSN-Gel-DOX platform in colon carcinoma cells, as well as *in vivo* where the as-developed nanoplatform showed significantly decreased tumor volumes in HT-29 xenografts when compared to DOX alone. Importantly, MSN-Gel-DOX depicted lower systemic toxicity in mice compared to free DOX administration, highlighting the advantage of rationally-designed nanomedicine approach over conventional chemotherapy.

**Figure 2:**
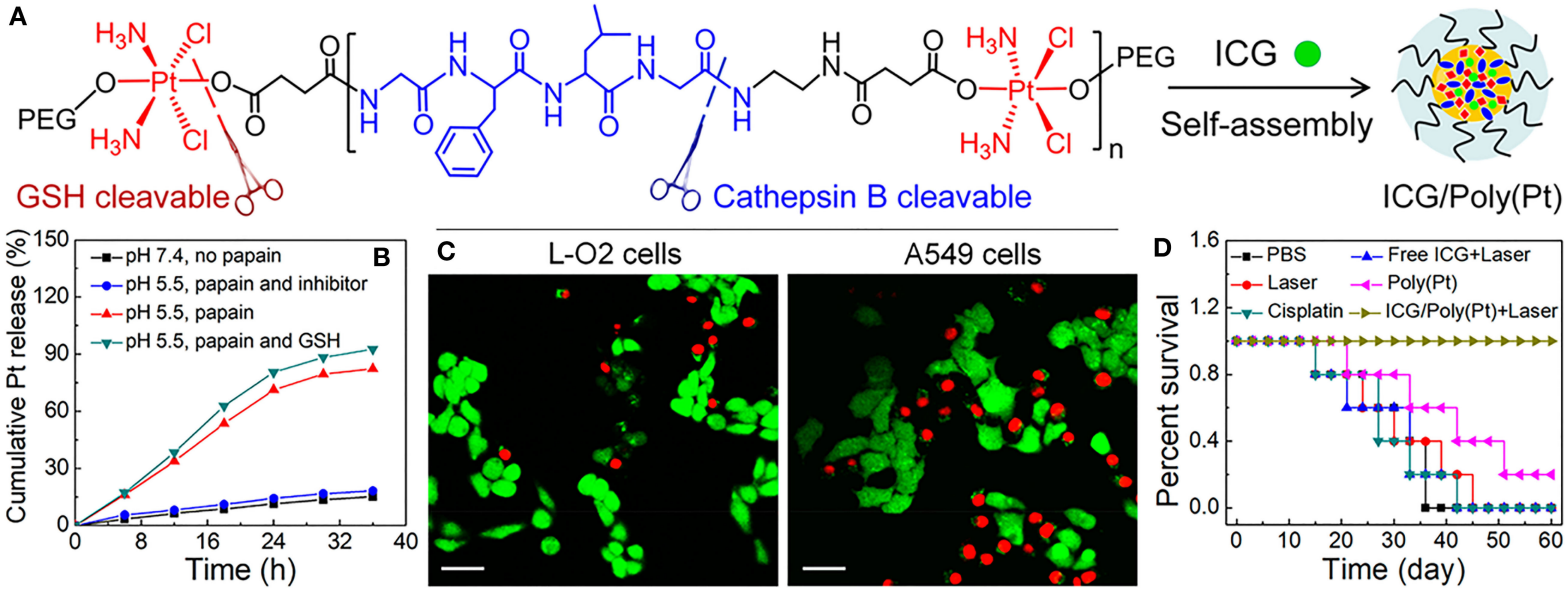
**(A)** Schematic illustration of Enzyme-Responsive Polyprodrug Nanoplatforms, composed of ICG and cisplatin polyprodrug amphiphiles tethered with repeating cathepsin-B degradable GFLG peptides, PEG-*b*-P(Pt-*co*-GFLG)-*b*-PEG. **(B)** *In vitro* Pt release under different treatment conditions: (i) pH 7.4 without papain (physiological condition), (ii) pH 5.5 with papain, simulating the acidic and enzymatic condition of lysosomes, (iii) pH 5.5 with papain in the presence of a papain inhibitor, and (iv) pH 5.5 with papain followed by GSH treatment at pH 7.4, mimicking the lysosomal escape into the reductive cytosolic environment. **(C)** Fluorescence images of cathepsin B positive A549 cells and cathepsin B negative L-O2 cells upon incubation with ICG/Poly(Pt) after irradiation with 808 nm, 1 W/cm^2^, 5 min. Viable cells were stained with green Calcein-AM, while dead and late apoptotic cells were stained with red PI. Scale bar: 40 um. **(D)** Survival curves of resistant A549/DDP cancerous mice after cascade photo-chemotherapy. Figure adapted with permission from Wang W. et al. (2018).

Highlighting the possibility for multimodal agents, Wang et al. developed a cisplatin polyprodrug nanoplatform for cascade photo-chemotherapy, through co-assembly of near infrared dye, indocyanine green (ICG) and polyethylene glycol (PEG) moieties, with repeating cathepsin-B degradable peptides and cisplatin prodrug units [ICG/Poly(Pt); Figure 2A] (Wang W. et al., 2018). Cathepsin B is a cysteine protease usually present in lysosomes that has been shown to be present in increased levels in tumors, particularly those that are metastatic and invasive (Gondi and Rao, 2013). In the paper noted, upon exposure to cathepsin B, the nanoplatform was degraded, resulting in the cascade chemotherapy beginning with release of ICG and cisplatin prodrug (Figure 2B). Further, irradiation with 808 nm light resulted in formation of ROS and hyperthermia for phototherapy (mediated by ICG) which promoted subsequent uptake of the cisplatin prodrugs to the cytosol and resulted in enhanced apoptosis in cathepsin B positive A549 cells (Figure 2C) *in vitro*. Enzyme-responsive treatment efficacy was also observed *in vivo*, where ICG/Poly(Pt) + laser treatment demonstrated higher survival rates in in resistant A549/DDP mice compared to untreated controls, or mice treated with only Pt, only cisplatin, or free ICG+laser (Figure 2D).

A major concern for enzyme-responsive treatment is the heterogenous expression of target enzymes in different cancer types. As the exploration of the TME continues, a better understanding of the expression patterns of enzymes at tumor sites will enable effective and precise enzyme-responsive drug delivery systems. It is also expected that newer and more universally expressed enzymes may discovered that may be exploited by nanotechnology for drug delivery. For a deeper overview of the current knowledge and future perspectives in the field, readers are directed to these excellent reviews on enzyme-stimulated drug delivery systems (de la Rica et al., 2012; Mu et al., 2018).

### ROS-Responsive Nanosystems

The physiology of the tumor site is notable also for the elevated presence of ROS, a byproduct of several physiological processes such as oncogene activation, metabolism, and mitochondrial dysfunction, that has been associated with abnormal cancer cell growth (Trachootham et al., 2009). Interestingly, NPs may also be used to selectively increase the ROS concentration within the tumor site to a level toxic to the cells, though this is not a focus of our discussion of ROS-mediated therapy (Ji et al., 2019; Kong et al., 2020). Rather, we focus here on endogenous ROS sensitivity. Several types of NPs have been recognized as promising for treatment of ROS-related diseases, most notably cerium oxide, carbon, and manganese NPs (Ferreira et al., 2018). Of these, ceria (cerium dioxide) NPs have been the most widely explored for cancer owing to their biocompatibility and antioxidant behavior (Wason and Zhao, 2013).

Endogenous ROS exploitation for drug delivery has been achieved through modifying MSNs with hydrophobic phenyl sulfide (PHS) moieties which protect the nanopores from being wetted by water and thereby inhibits premature release of drugs, such as DOX. Conversely, under the stimulation of endogenous ROS, the PHS groups are oxidized and the nanopores are wetted, resulting in enhanced DOX release (Cheng et al., 2017). Although confined to *in vitro* studies only, the system represents an excellent example of nanoengineering approaches to design simple but effective stimuli-responsive drug delivery nanomedicines. ROS-responsive NPs have also been designed by exploiting thioketal (TK) containing linkers in an elegant study by Xu et al. (2017b). The authors designed a polyprodrug platform from a model drug mitoxantrone (MTO) polymerized with TK linkers and polyethylene glycol. Further tumor specificity was endowed via integrin-targeting RGD ligand. Self-assembled NPs demonstrated enhanced MTO delivery and tumor inhibition *in vivo* in LNCaP prostate tumors, known to have high ROS concentration, compared to free MTO drug, as well as NPs without ROS-sensitive TK linkers (Xu et al., 2017b).

Since the levels of ROS change with tumor status, an innovative strategy was used to combine chemotherapeutic DOX and photodynamic agent Ce6 in polymeric NPs, capable of *in situ* ROS generation and enhanced anti-cancer therapy (Cao et al., 2018). When irradiated with 660 nm laser, ROS generated by the activation of Ce6 resulted in cleavage of the TK linker, causing shrinkage of the NP backbone and enhanced release of DOX. Compared to control, the treatment groups demonstrated remarkable therapeutic outcomes *in vivo*, highlighting the promising potential of remotely-controlled light-activated targeted drug delivery systems. Notably, TK-PPEs administered without internalized Ce6 did not demonstrate ROS-responsiveness, possibly owing to the lower intrinsic ROS levels in the tumor. The approach was mirrored by another group, who reported TK linked, PEGylated NPs with a Ce6 photosensitizer loaded with paclitaxel (PTX; TK-Ce6-PTX NPs) (Li et al., 2018). *In vivo* studies revealed prolonged circulation time of PEGylated NPs. TK-Ce6-PTX NPs with laser irradiation showed increased tumor tissue concentration of PTX compared to TK-NP-Ce6-PTX-NPs without irradiation, and had little PTX uptake in off-target organs. Overall, this strategy overcomes tumor heterogeneity and can be effective for highly targeted ROS-mediated multimodal therapy in tumors that typically demonstrate low ROS concentrations.

### Hypoxia-Responsive NPs

Hypoxia is an important biomarker of aggressive tumors, which is widely associated with poor clinical outcomes for the three pillars of cancer treatment (Harris, 2002). Hypoxia is a regulator of numerous pathways that are critical to tumor development and maintenance, such as angiogenesis and metastasis, though it may also induce cell death by apoptosis (Harris, 2002). Resulting from accelerated metabolism or deficient oxygen delivery, hypoxic TME provides a reducing environment with increased presence of nitroreductases and azoreductases (Cui et al., 2011; Liu et al., 2017). Thus, targets of these species such as nitroaromatic, quinone, and azobenzene derivatives have been exploited as hypoxia-sensitive moieties in the development of TME-responsive nanomedicine (Cui et al., 2011; Liu et al., 2017). For example, hypoxia-activated prodrugs (HAPs), which are non-toxic compounds designed to undergo reduction to cytotoxic compounds in the hypoxic environment, have been widely reported for cancer therapy (Hunter et al., 2016). A number of HAPs have been interrogated in clinical trials with limited efficacy that has been attributed to various factors, including poor extravascular transport and suboptimal micropharmacokinetic properties (Jackson et al., 2019). Thus nanocarriers have been designed to improve the accumulation of HAPs at the tumor sites (Liu et al., 2015a).

More innovative strategies have utilized endogenous hypoxia sensitivity as a trigger to enhance drug delivery. Son et al. reported a carboxymethyl dextran (CMD) NP containing a hypoxia-sensitive azo moiety as well as black hole quencher 3 (BHQ3) dye (Son et al., 2018). Under hypoxic conditions, CMD-BHQ3-NPs were reduced to aniline derivative by tumor intrinsic reductases. These NPs were also loaded with DOX and demonstrated increased drug release in hypoxic conditions compared to normoxic conditions. *In vivo*, the NPs showed high tumor accumulation, demonstrating potential for the system to be used for hypoxia-induced drug delivery. Later, a nanosystem was synthesized with a nitro-imidazole derivative conjugated to CMD, forming hypoxia-responsive NPs (HR-NPs) that were loaded with DOX (Thambi et al., 2014). On exposure to hypoxic conditions, the nitro-imidazole group of the HR-NPs was reduced to aminoimidazole, successfully demonstrating hypoxia-responsiveness, and the NPs showed increased DOX release compared to NPs in the normoxic condition. *In vivo* studies showed high accumulation of the HR-NPs in the tumor site, and slowed tumor growth compared to mice treated with saline or free DOX. The use of a nitroimidazole derivative to create hypoxia-responsiveness has also been seen elsewhere (Ahmad et al., 2016).

Recently, increased attention has been paid to the potential of combination of PDT and hypoxia-responsive nanoplatforms (Qian et al., 2016), again highlighting the importance of multifaceted nanomedicine that can target one tumor in many ways to improve therapeutic outcomes. Along these lines, there is room for hypoxia-responsive or hypoxia-alleviating NPs that can also result in enhanced ERT, which is known to be adversely affected by hypoxia(also discussed further in the next section) (Rockwell et al., 2009). Using hypoxia-sensitive NPs followed by hypoxia alleviating or radiosensitizing NPs may present an effective strategy for multipronged attack on the tumor site in future explorations.

A consideration for all stimuli-responsive systems discussed thus far, is the ability of the NP to maintain the full dose of drug with which it is loaded during its transport to the tumor site, and release the full dose upon exposure to the TME. In other words, NPs need to maintain their specificity for only TME triggers, and respond only when necessary, to avoid undesirable side effects which have become a hallmark of traditional chemotherapy. As our knowledge of tumor physiology as well as tumor vasculature and how it relates to the TME triggers improves, so does the potential for improvement in stimuli-responsive nanomedicine.

## Nanotechnology for Enhancing External Radiation Therapy (ERT)

ERT is one of the long-standing pillars of cancer therapy, performed either alone in cases where surgery is not possible, or in conjunction with surgery or chemotherapy. Adjuvant radiotherapy is a standard in clinical care, whereby residual tumor margins after debulking surgery are irradiated to prevent recurrence and relapse (Coffey et al., 2003). ERT utilizes high energy ionizing beams to directly target the tumor site (Haume et al., 2016). This poses short term risks such as skin irritation as well as long term risks such as fibrosis and atrophy to nearby healthy tissue (Bentzen, 2006). In addition to this complication, there is evidence to show that hypoxic areas within the tumor site are more resistant to standard ERT than non-hypoxic areas (Rockwell et al., 2009). Nanotechnology can play an integral role in improving radiotherapy through improved treatment delivery, combination with other treatment modalities and companion diagnostics (Erdi et al., 2002).

**Figure 3:**
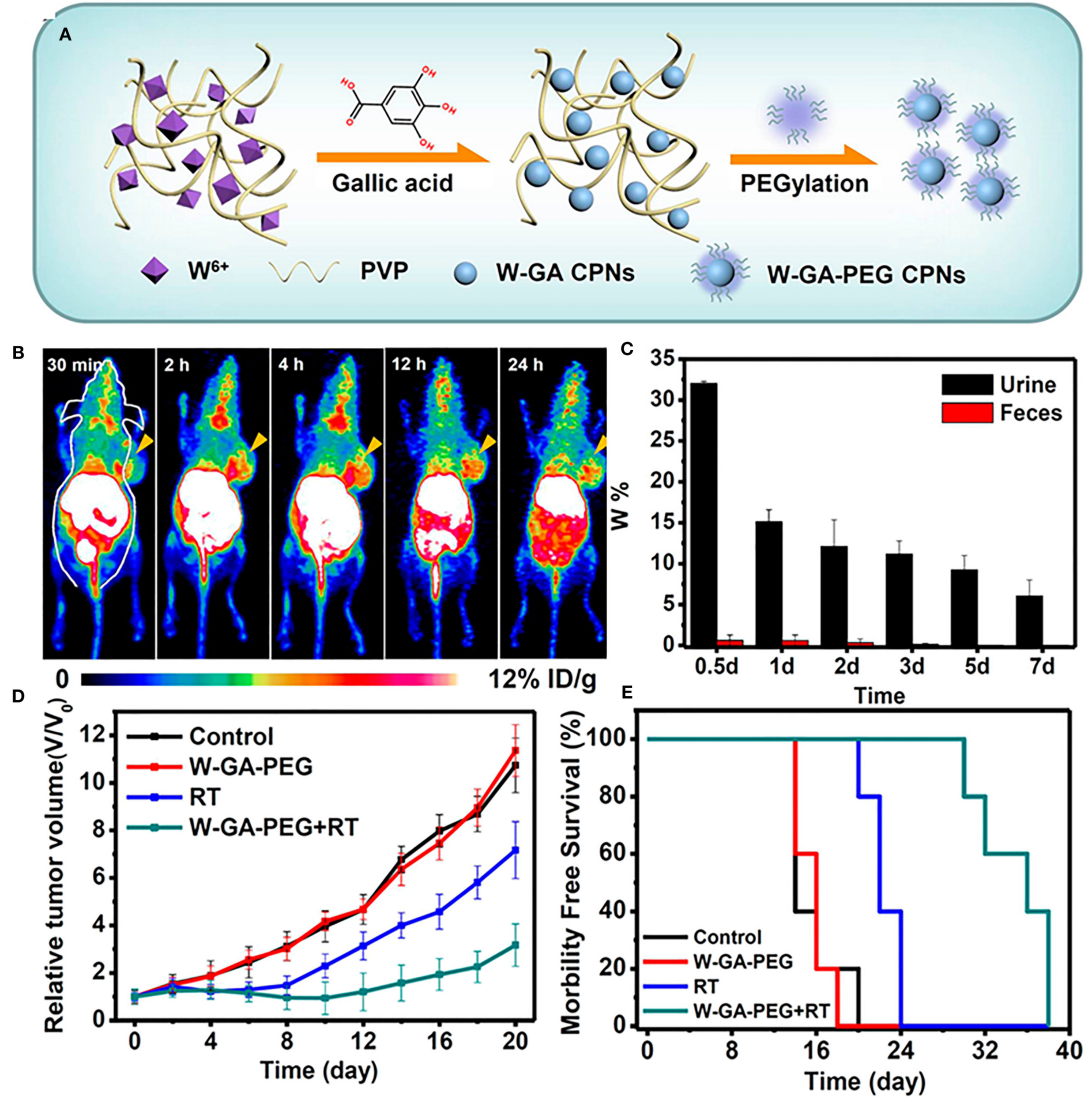
**(A)** Schematic illustration for the synthesis and structure of W-GA-PEG CPNs. **(B)** *In vivo* PET images of 4T1-tumor-bearing mice after i.v. injection of 64Cu-W-GA-PEG CPNs at different time points. The tumors are indicated with yellow arrows. **(C)** The W levels in urine and feces of healthy mice after i.v. administration of W-GA-PEG CPNs (dose = 40 mg/kg) collected at different intervals. **(D)** Tumor growth curves and **(E)** survival curves of animals treated with W-GA-PEG CPN or radiotherapy or both (five mice per group; irradiation dose of X-ray (RT): 6 Gy; injection dose of W-GA-PEG CPNs: 20 mg/kg). Figures adapted from Shen et al. (2017).

Nanomaterials with high photoelectric cross-sections have been shown to amplify effective radiation dose locally at the tumor site, thereby significantly reducing unwanted side effects and overexposure to radiation (Goel et al., 2017). In an noteworthy study, Shen et. al. reported a biocompatible, renal-clearable nanosystem composed of PEGylated tungsten-gallic acid coordination polymers (W-GA-PEG CPNS) ~5 nm in diameter (Figure 3A) (Shen et al., 2017). ^64^Cu-labeled W-GA-PEG CPNS demonstrated significant uptake in 4T1 tumor bearing mice within 4 h post-injection, as revealed by positron emission tomography (PET, Figure 3B) along with rapid renal clearance and little long-term retention (Figure 3C). Mice treated with W-GA-PEG CPN combined with RT demonstrated significantly reduced tumor volumes and prolonged survival compared to mice treated with RT alone (Figures 3D,E). Given that many of the nanoplatforms researched for combination with RT involve heavy metals, this study addresses a major concern in improving biocompatibility and natural clearance to avoid long term toxicity in cancer treatment. Nanoformulations have also been designed to improve the delivery of radiosensitizers such as Wortmannin, a potent inhibitor of DNA-dependent kinases, limited by its insolubility and poor pharmacokinetic profule (Karve et al., 2012). Uses of nanomaterials in radiosensitization have been widely explored, becoming the subject of several reviews (Kwatra et al., 2013; Mi et al., 2016; Goel et al., 2017; Song et al., 2017).

Another relevant target for NP intervention to improve ERT is modulating the hypoxic center that exists within solid tumor sites, as was alluded to previously (Goel et al., 2017; Graham and Unger, 2018). Anti-cancer effect of ionizing radiations is dependent on the generation of ROS, which is turn depends of the oxygen availability in the TME. Thus, hypoxic centers cause tumor cells to become radiation-resistant and decrease the efficacy of ERT (Moulder and Rockwell, 1987). Nanotechnology has enabled a two-pronged attack on this issue: nanocarriers loaded with hypoxia-activated drugs have been designed for combinatorial chemo-radiation therapies. A recent noteworthy study reported a rattle-type nanostructure comprised of an upconversion nanoparticle core with a mesoporous silica shell (UCHM) (Liu et al., 2015b). UCHMs loaded with the hypoxia-sensitive agent tirapazamine (TPZ) demonstrated complete tumor remission when combined with RT. On the other hand control mice treated with RT or TPZ alone saw slowed tumor growth initially, which accelerated significantly over time. This effect was also seen in the mice treated with UCHMs and RT, while groups treated with TPZ and RT saw significantly decreased tumor growth.

Nanomaterials have also been engineered to alleviate hypoxia and reoxygenate the tumor microenvironment through enhanced oxygen delivery (Zhou Z. et al., 2018) or *in situ* oxygen production (Prasad et al., 2014). An example of the latter strategy comes from Prasad et al. who developed manganese dioxide (MnO_2_) nanoparticles conjugated with albumin that react with endogenous H_2_O_2_ to produce O_2_, with the beneficial side effect of increasing the local pH to combat tumor acidosis, previously mentioned as a hallmark of the TME (Prasad et al., 2014). When injected intratumorally into EMT6 tumor bearing mice, the MnO_2_-Albumin NPs caused 45% increase in saturated O_2_ levels at tumor periphery compared to mice without NP treatment. In the future, the ability of NPs to alleviate the hypoxic center may be employed to sensitize the tumor to lower doses of radiation, so as to achieve an enhanced treatment with even further reduced side effects.

The ability to extend beyond the three pillars and combine different therapies into one system is one of the biggest advantages that nanomedicine brings to traditional treatment regimes. In an exciting study, nanoscale metal-organic frameworks (nMOFS) were harnessed to combine radiotherapy-radiodynamic therapy and immune checkpoint blockade for local and systemic tumor elimination (Lu et al., 2018). The authors synthesized hafnium nMOFS, which absorbed X-Ray photons that directly excited a coordinated porphyrin photosensitizer. This resulted in both radiotherapy and the production of singlet oxygen species for enhanced radiodynamic therapy even at low doses. To achieve dual radiotherapy and immunotherapy, the nMOFs were loaded with an inhibitor of indoleamine 2,3, dioxygenase, an immunoregulatory enzyme establishes immune tolerance in the TME. The study ultimately demonstrated *in vivo* regression of primary tumors as well as untreated distant tumors via abscopal effect. Importantly, the authors demonstrated systemic tumor rejection when re-challenged, indicating the potential of X-radiation induced *in situ* vaccination. Overall, this study serves as a key example of innovative and effective nanomedicines that synergistically combine multiple pillars of cancer therapy with promising outcomes.

## Nanomedicine Approaches in Immunotherapy

The immune system has limited natural ability to fight cancer, and the TME is often marked by immunosuppressive and immune-evasive mechanisms (Couzin-Frankel, 2013). Immunotherapy seeks to re-train the body's immune system to recognize cancer as non-self and appropriately respond, without triggering undesirable autoimmune processes (Pardoll, 2012). There are several described ways of initiating immunotherapy, including T cell priming and therapy (Schirrmacher et al., 2003), antigen release, and checkpoint inhibition (Pardoll, 2012), and nanotechnology has interacted with all of these domains (Liu et al., 2018; Goldberg, 2019; Irvine and Dane, 2020).

NPs have been used to deliver immunostimulatory agents, antigens, cytokines, chemokines, nucleotides, and toll-like receptor (TLR) agonists that target various immune cells (Fan and Moon, 2015; Da Silva et al., 2016). In other cases, NP design has been carefully modulated to help mount anti-tumor immune responses through their material compositions, geometries, or surface modifications (Moon et al., 2012; Wang J. et al., 2018).

In cases where adaptive immune responses cannot be mounted, nanomedicine approaches have been employed to trigger the innate immune system. In this regard, cancer nanovaccines and more recently nanotherapies activating anti-tumor phagocytes (macrophages) and NK cells are particularly noteworthy (Yuan et al., 2017). For example, a multivalent nanobioconjugate engager (mBiNE) was developed to simultaneously target human epidermal growth factor 2 (HER2), which is overexpressed in certain breast cancers, and calreticulin, a prophagocytic protein (Yuan et al., 2017). The mBiNe led to enhanced phagocytosis of cancer cells and enhanced antigen presentation by macrophages in HER2 positive cells. *In vivo* studies showed that mBiNE had enhanced antitumor efficacy in HER2 positive tumors compared to HER2 negative tumors. Treatment with mBiNE led to increased presence of macrophages and T cells in the tumor site. Interestingly, mice treated with mBiNE also demonstrated resistance to re-challenge and enhanced antitumor immunity in both HER2 negative and HER2 positive tumors. Further, cancer nanovaccines based on dendritic cells (DC vaccines) and NK cells have also shown significant promise in preclinical studies. The development of nanovaccines is a highly active area of research, covered extensively in excellent prior reviews (Irvine et al., 2015; Luo et al., 2017).

An immunotherapy strategy focusing on the adaptive immune system, immune checkpoint inhibition (ICI), has demonstrated excellent therapeutic outcomes and has now become a subject of intense research both in the clinical and preclinical settings (Alsaab et al., 2017). Among many targets, ICI may exploit the unusually high density of the protein, programmed death ligand (PD-L1) on the tumors, that orchestrates immune evasion by inhibiting cytotoxic lymphocyte (CTL) function. Anti-PD-L1 antibodies have shown significant clinical benefit in response rate, survival, and side effects, making it popular as of late (Alsaab et al., 2017). However, ICI has been shown to be effective in only a small subset of patients, and only against certain cancer types, calling for innovation (Alsaab et al., 2017). An interesting study utilized the PD-L1 checkpoint inhibition strategy along with an aldehyde-functionalized dextran superparamagnetic iron oxide NPs, which were conjugated with a checkpoint inhibitor and T cell activators. The NPs could be targeted to the tumor site using an external magnetic field, and once there, modulated the immunosuppressive environment by increasing T cell proliferation as well as ICI (Chiang et al., 2018). This created a twofold immune response that inhibited the tumor growth in different tumor models *in vivo*; 4T1 breast cancer and CT-26 colon cancer, providing a promising avenue for future research in nanotechnology and immunotherapy (Chiang et al., 2018). Nanotechnology may also combine delivery of PD-L1 antibodies with PDT for enhanced response, as was done with non-toxic core-shell NPs (Duan et al., 2016). Apoptosis/necrosis of tumor cells as well as disrupted vasculature after PDT increased the tumor immunogenicity by activating both the innate and adaptive immune systems in the TME. When combined with the PD-L1 blockade, not only localized but systemic antitumor response was successfully mounted in syngeneic breast cancer models (Duan et al., 2016).

**Figure 4:**
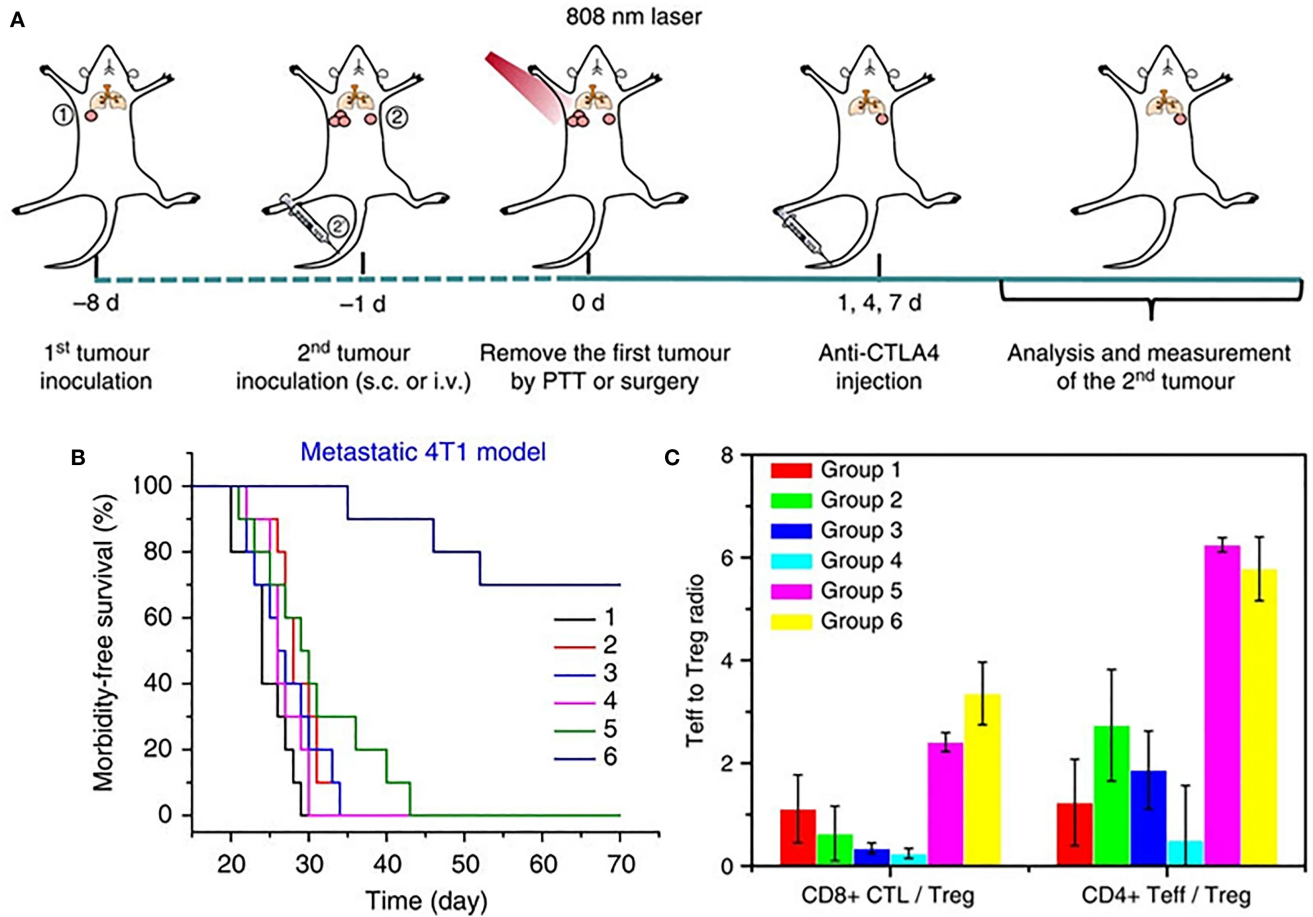
**(A)** Schematic illustration of PLGA-ICG-R837-based PTT and anti-CTLA-4 combination therapy to inhibit tumor growth at distant sites. **(B)** Morbidity-free survival of different groups of mice with metastatic 4T1 tumors after various treatments to eradicate their primary tumors: (1) Surgery, (2) Surgery+antiCTLA4, (3) Surgery+ PLGA-ICG-R837, (4) PLGA-ICG-R837+laser, (5) Surgery+ PLGA-ICG-R837+antiCTLA4, and (6) PLGA-ICG-R837+ laser + antiCTLA4. **(C)** CD8+ CTL: Treg ratios and CD4+ effector T cells: Treg ratios in the secondary tumors upon various treatments. Both ratios were significantly enhanced after combination treatment with PLGA-ICG-R837-based PTT and anti-CTLA4 therapy. Figure adapted with permission from Chen et al. (2016).

ICI targeting cytotoxic T-lymphocyte-associated antigen 4 (CTLA4) has also been thoroughly studied and enhanced by nanotechnology. Mechanism of action of CTLA4 is under intense research. CTLA4 has been shown to outcompete T cell co-stimulatory receptor, CD28 to bind CD80 and CD86 ligands, and creating an immunosuppressive environment which is conducive to tumor growth (Pardoll, 2012). It also sequesters CD80 and CD86 ligands from binding CD28 moeities on the surface of T cells, thus blocking the co-stimulatory signal required for T cell activation, as well as actively removing these ligands from APC surface. CTLA4 blockade serves to enhance CD4^+^ T cell activity as well as reverse the immunosuppressive environment normally maintained by an increased presence of T_reg_ cells. CTLA4-blocking antibodies have been an area of ongoing research and were the first class of immunotherapeutics to be FDA approved (Pardoll, 2012). However, as with other ICI strategies, CTLA4-based treatment shows heterogeneous responses. Recently, PLGA NPs combining ICG and the toll like receptor ligand R387 (PLGA-ICG-R387) were used in combination photothermal-immunotherapy, such that the photothermal ablation triggered the release of tumor-associated antigens, which in combination with anti-CTLA4 checkpoint blockade, resulted in strong immunological response both locally and at distant tumor sites (Figure 4A) (Chen et al., 2016). This response led to increased DC maturation *in vitro* and *in vivo*, as well as demonstrated slowed growth of off-target tumors (Figure 4B). In a long-term immune-memory study, secondary tumors that were re-inoculated 40 days after treatment with the PLGA-ICG-R387 and ablation, showed increased levels of T-cells, IFN-δ, and TNF-α and delayed tumor growth compared to tumors that had been re-inoculated 40 days post-surgery alone (Figure 4C). This, along with other notable work using upconversion NPs triggered by NIR light to combine CTLA4 checkpoint blockade with PDT (Xu et al., 2017a), shows the potential for multifunctional NPs to effectively consolidate treatment options while delivering improved therapeutic outcomes.

The use of immunotherapy in cancer treatment is a recent and exciting development. Much is left to be discovered about the role of the natural immune system in preventing, managing, and fighting cancer. As more is learned about immunotherapeutics, the uses of nanotechnology in the domain will almost certainly evolve beyond those discussed here, especially with regards to innate immunity and nanovaccines. Further, nanotechnology may allow for more streamlined combination of immunotherapy with other modalities such as chemo or radiation therapies as well as image-guided stratification of immunologically “hot” and “cold” tumors, all of which can work together to improve patient outcomes in the future.

## Conclusions and Future Perspectives

**Figure 5:**
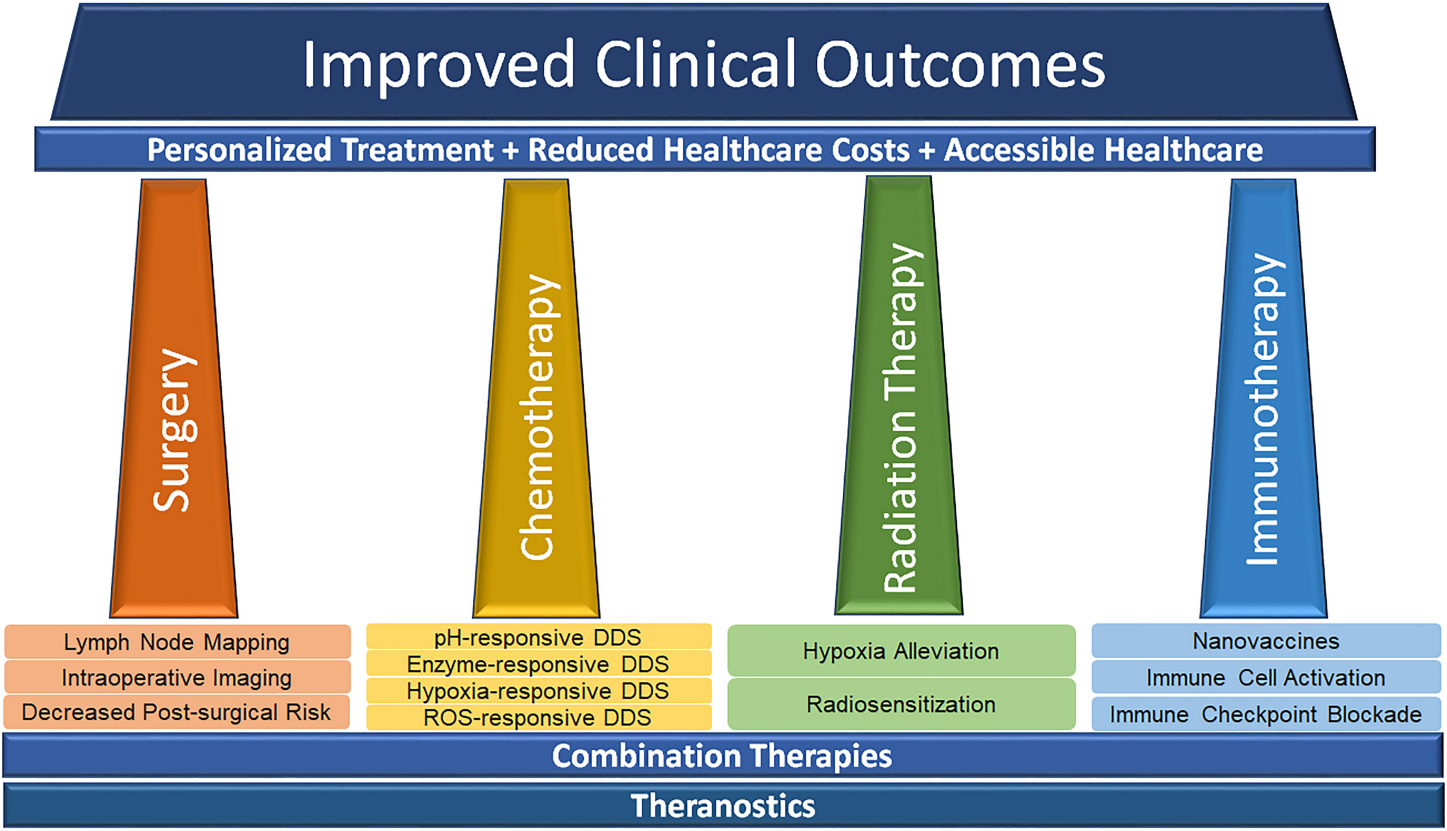
Schematic illustrating the potential of nanotechnology in advancing the pillars of modern cancer treatment toward effective, affordable and accessible personalized medicine to improve healthcare outcomes.

The level of innovation demonstrated by nanotechnology, as applied to the pillars of cancer treatment has been phenomenal in the preclinical arena. As we have covered here, nanotechnology both has the ability to improve the pillars individually, as well as facilitate combination therapies with the ultimate goal of improving clinical outcomes (Figure 5). Translation of these novel strategies to the clinic, however, has not progressed in accordance with the literature. In the clinical domain, this innovation has been conspicuous by its absence, as doxil and abraxane continue to dominate the clinical utility of nanotechnology, accounting for > $1 B dollars in sale annually (Grodzinski et al., 2019). While these figures are impressive, there is much work to improve, particularly when the balance of efficacy and safety are concerned. In terms of chemotherapy and targeted drug delivery, while NPs have already been used for drugs that are generally hydrophobic or otherwise display poor pharmacokinetics *in vivo*, there is room for repurposing drugs that have been previously rejected (or orphan drugs). If appropriately formulated, NPs show significant promise in making them viable options for treatment in the clinic.

As nanotechnology progresses from the research setting to the clinical one, attention must be paid to the toxicity of not only drugs themselves, but the delivery systems which are being developed. Clinical translation of nanosystems depends on their stability in circulation, ability to negotiate physiological barriers to access the tumor site and their safety profile. This latter point has significantly impeded the clinical successes of nanomedicine so far. As such, NPs which can be cleared naturally by the body or which degrade after treatment are desirable. It is important to thoroughly characterize and deconstruct nanoparticle transport and toxicity not only in the short term, but also long term. Continued progress of nanofabrication methodologies provides the potential for incorporating imaging labels onto therapeutic nanomaterials to develop modular designs that enable non-invasive delineation of nanoparticles kinetics *in vivo* in real time (Goel et al., 2020). Better understanding of NP transport in different animal models over longer timescales would function not only to improve treatment outcomes, but also to help anticipate long term off-target side effects during translational studies. Avoiding cumulative buildup of NPs in the body is a crucial long term consideration that remains an important hurdle to overcome prior to nanomedicines becoming clinically and commercially viable. Similar considerations are paramount in the use of nanotechnology for radiation therapy.

Cancer immunotherapy is a rapidly evolving and highly promising area of research, with great potential for improvement with nanotechnology. NPs may be used in numerous contexts encompassing both innate and adaptive immunity, as well as potential cancer vaccines. The combination of immunotherapy and other therapies such as chemo or radiation therapies facillitated ny nanotechnology may not only increase the efficacy of treatment and overcome innate immunological “coldness” of certain tumors, but also lead to more convenient administration in the clinic. As immunotherapy grows, more emphasis may be placed on individualized therapies, and the ability to potentially combine a more general chemotherapeutic treatment with an individualized immunotherapy could be exciting, both weakening the tumor and specifically strengthening the host's immune response. Furthermore, given the systemic nature of immunomodulatory therapies, particularly cancer nanovaccines that are trafficked through the lymphoid structures, it is essential that thorough biodistribution studies are performed at both organ and cellular levels. As such, the role of integrated multiscale imaging methods is indispensable.

Finally, the versatility of nanotechnology in cancer requires concerted efforts and interdisciplinary cooperation between scientists, academics, clinicians and regulatory authorities. Continued support from funding agencies and improved cross-talk between academia and industry will be essential to move cancer nanomedicine forward. While significant attention is paid to improving nanomedicine design, it is equally important to design rigorous clinical trials based on appropriate patient selection and stratification, as well as identification of unique avenues in cancer treatment that will benefit from integration of nanotechnology.

## Author Contributions

CS and SG co-wrote the article. SG and WC contributed to planning and guidance. All authors approve the submitted version.

## Conflict of Interest

The authors declare that the research was conducted in the absence of any commercial or financial relationships that could be construed as a potential conflict of interest.
